# Supplementary material for: Folliculin promotes substrate-selective mTORC1 activity by activating RagC to recruit TFE3
Source: PLoS Biol. 2022 Mar 31;20(3):e3001594. doi: 10.1371/journal.pbio.3001594 (PMC9004751; doi:10.1371/journal.pbio.3001594)
Supplement: S1 Fig — (A) Control C2C12 cells (NTC) or cells lacking Flcn, Tsc2, or Depdc5 were switched from complete medium to media lacking serum and/or AAs for 60 minutes followed by immunoblotting. Images were uploaded into ImageJ, and signal intensity was quantified. Graphed above is the ratio of p-TFE3 to total TFE3 signal. (B) Quantification and graph of ratio of pS6K to total S6K signal. The data underlying all the graphs shown in the figure is included in S1 Data. AA, amino acid; FLCN, folliculin. (PDF) [file pbio.3001594.s001.pdf]

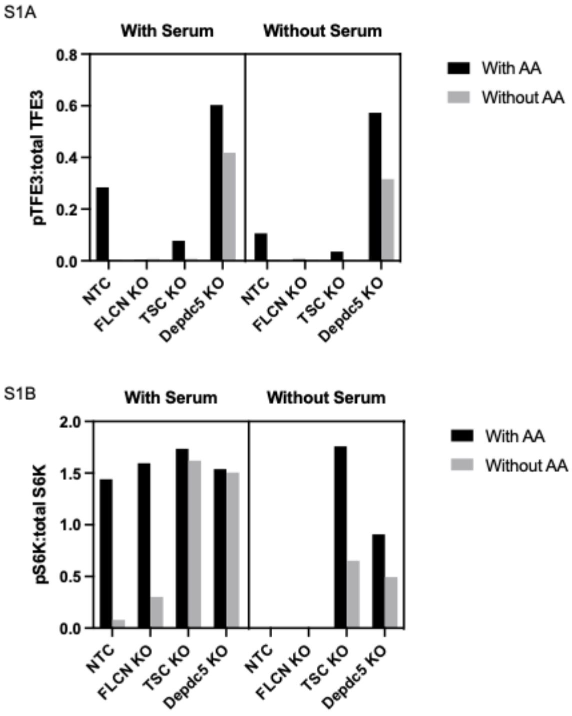

(S1) TFE3 phosphorylation is responsive to amino acids, via the GATOR complex. (A) Control C2C12 cells (NTC) or cells lacking Flcn, Tsc2, or Depdc5, were switched from complete medium to media lacking serum and/or amino acids for 60 minutes followed by immunoblotting. Images were uploaded into ImageJ and signal intensity was quantified. Graphed above is the ratio of p-TFE3 to total TFE3 signal. (B) Quantification and graph of ratio of pS6K to total S6K signal. The data underlying all the graphs shown in the figure is included in the S1 Data file.
