# Supplementary material for: Folliculin promotes substrate-selective mTORC1 activity by activating RagC to recruit TFE3
Source: PLoS Biol. 2022 Mar 31;20(3):e3001594. doi: 10.1371/journal.pbio.3001594 (PMC9004751; doi:10.1371/journal.pbio.3001594)
Supplement: S2 Fig — (A) Coexpression of active RagA and RagC in 293T cells does not confer further phosphorylation of TFE3 compared to active RagC alone. (B) RagC 75L protein is partially stabilized by inhibition of the proteasome with MG132. (C) To accompany main Fig 2C, immunoblotting for total levels of TFE3, S6K1, and 4E-BP in C2C12 cells expressing HA-tagged WT, or constitutive active RagA (GTP) or RagC (GDP), demonstrate equivalent expression of these proteins at all time points after switching from complete medium to media lacking AAs. AA, amino acid; WT, wild type. (PDF) [file pbio.3001594.s002.pdf]

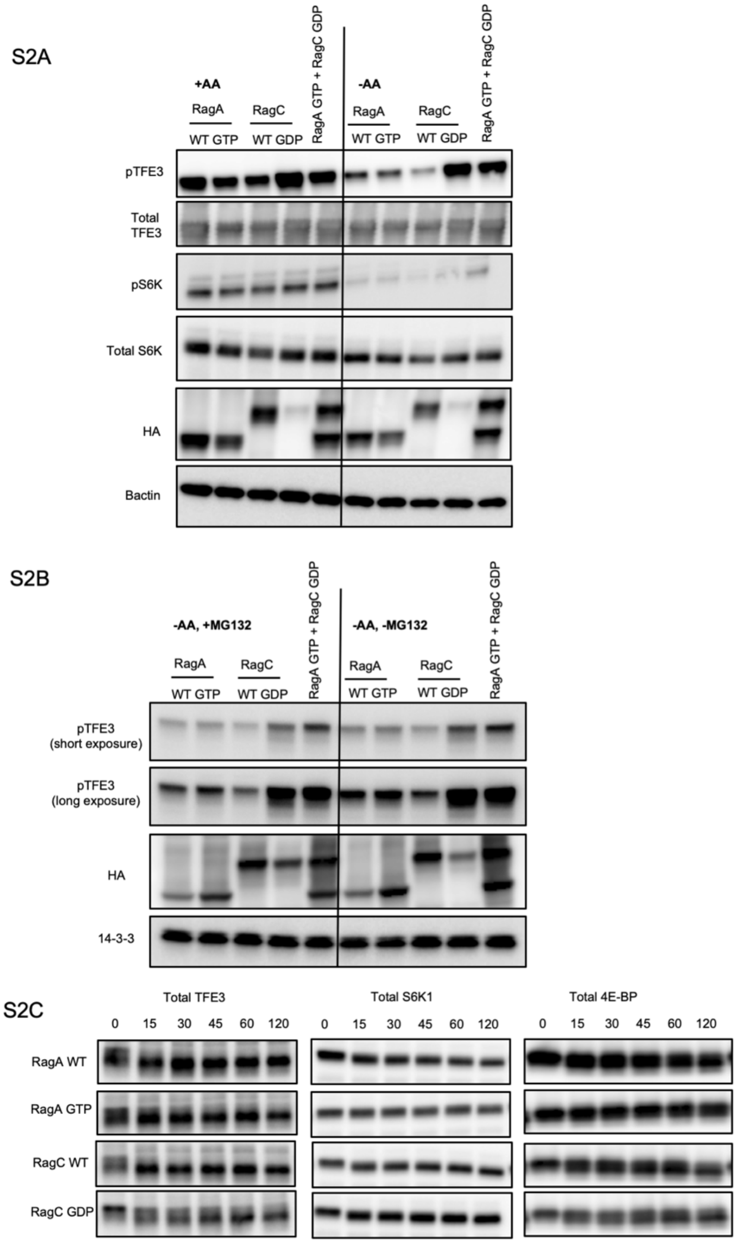

(S2) RagC, but not RagA, promotes TFE3 phosphorylation in response to AAs. (A) Co-expression of active RagA and RagC in 293T cells does not confer further phosphorylation of TFE3 compared to active RagC alone. (B) RagC 75L protein is partially stabilized by inhibition of the proteasome with MG132. (C) To accompany main Fig 2C, immunoblotting for total levels of TFE3, S6K1, and 4E-BP in C2C12 cells expressing HA-tagged wildtype (WT), or constitutive active RagA (GTP) or RagC (GDP), demonstrate equivalent expression of these proteins at all time points after switching from complete medium to media lacking amino acids.
