# Supplementary material for: Folliculin promotes substrate-selective mTORC1 activity by activating RagC to recruit TFE3
Source: PLoS Biol. 2022 Mar 31;20(3):e3001594. doi: 10.1371/journal.pbio.3001594 (PMC9004751; doi:10.1371/journal.pbio.3001594)
Supplement: S3 Fig — (A) C2C12 cells with RagC CRISPR KO were used for Fig 5A. (B) RagC CRISPR KO (in C2C12s) and RagC siRNA knockdown (in HEK 293Ts) showed no significant compensation of RagD expression. The data underlying all the graphs shown in the figure is included in S1 Data. KO, knockout. (PDF) [file pbio.3001594.s003.pdf]

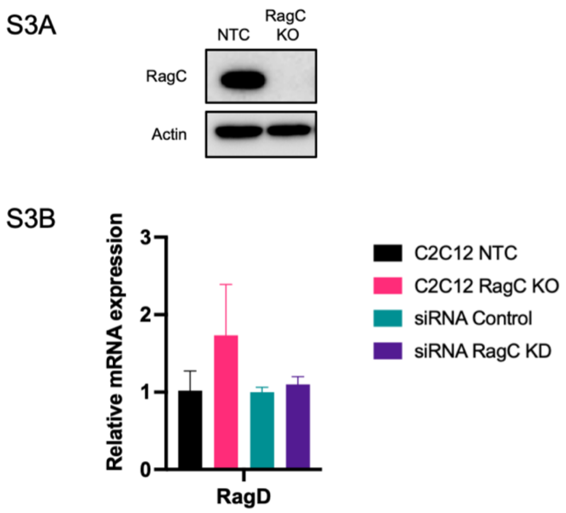

(S3) RagD expression is not increased as compensation for RagC CRISPR knockout or siRNA knockdown. (A) C2C12 cells with RagC CRISPR knockout were used for Fig 5A. (B) RagC CRISPR knockout (in C2C12s) and RagC siRNA knockdown (in HEK 293Ts) showed no significant compensation of RagD expression. The data underlying all the graphs shown in the figure is included in the S1 Data file.
