# Supplementary figures and images for: Folliculin promotes substrate-selective mTORC1 activity by activating RagC to recruit TFE3
Source: PLoS Biol. 2022 Mar 31;20(3):e3001594. doi: 10.1371/journal.pbio.3001594 (PMC9004751; doi:10.1371/journal.pbio.3001594)

Fig 1A

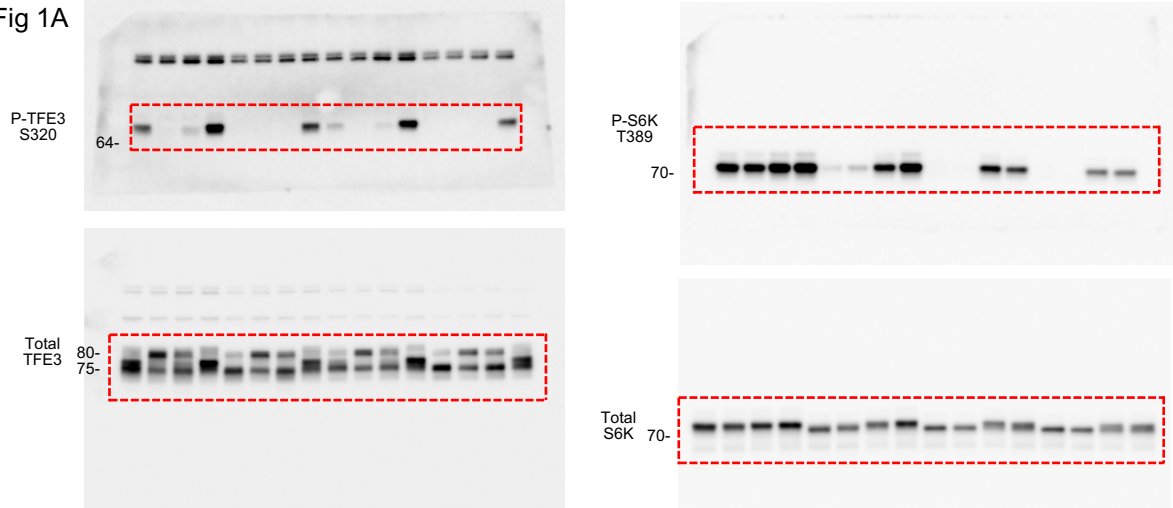

Fig 1C

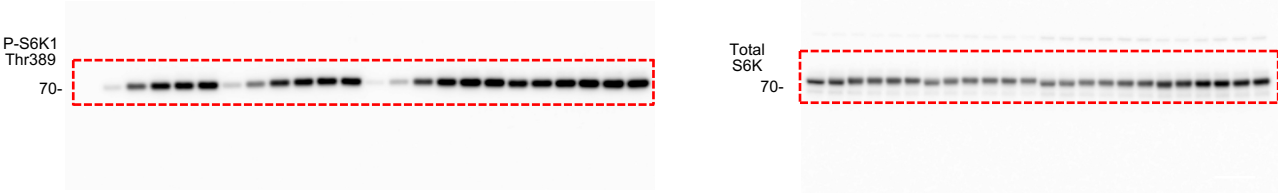

Fig 1D

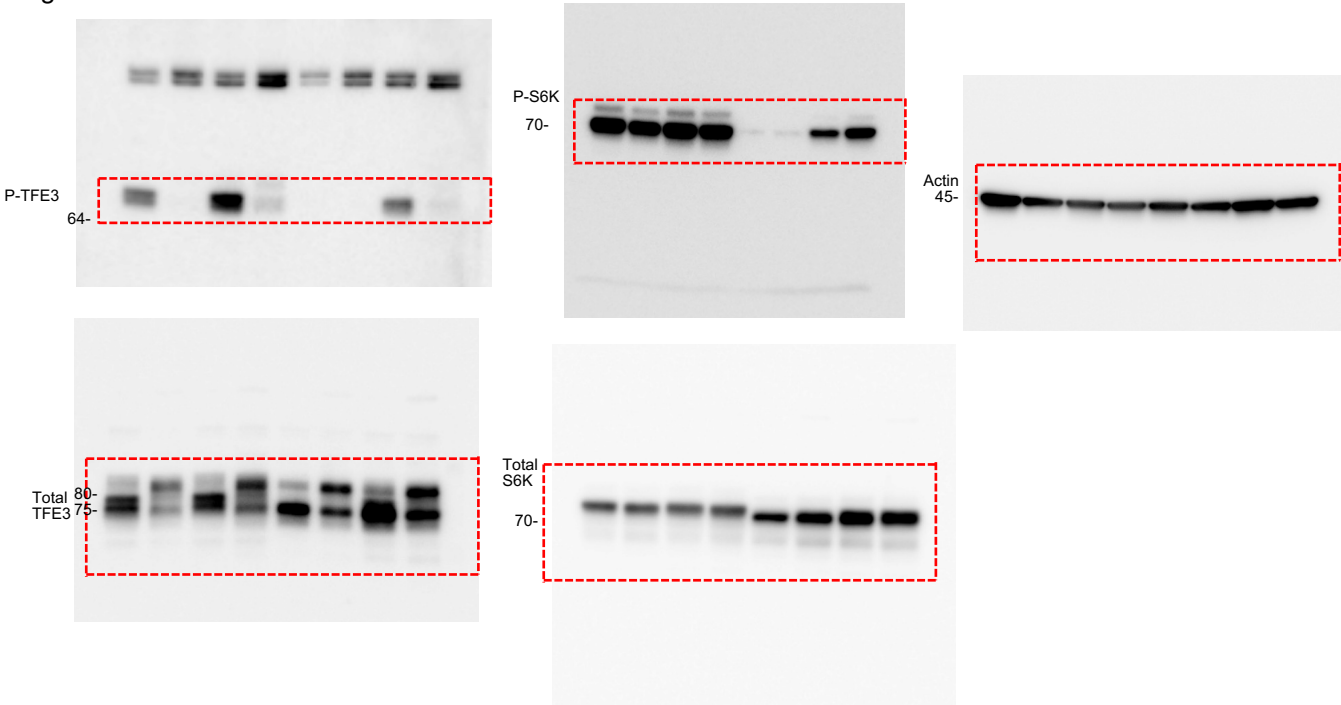

Fig 2A

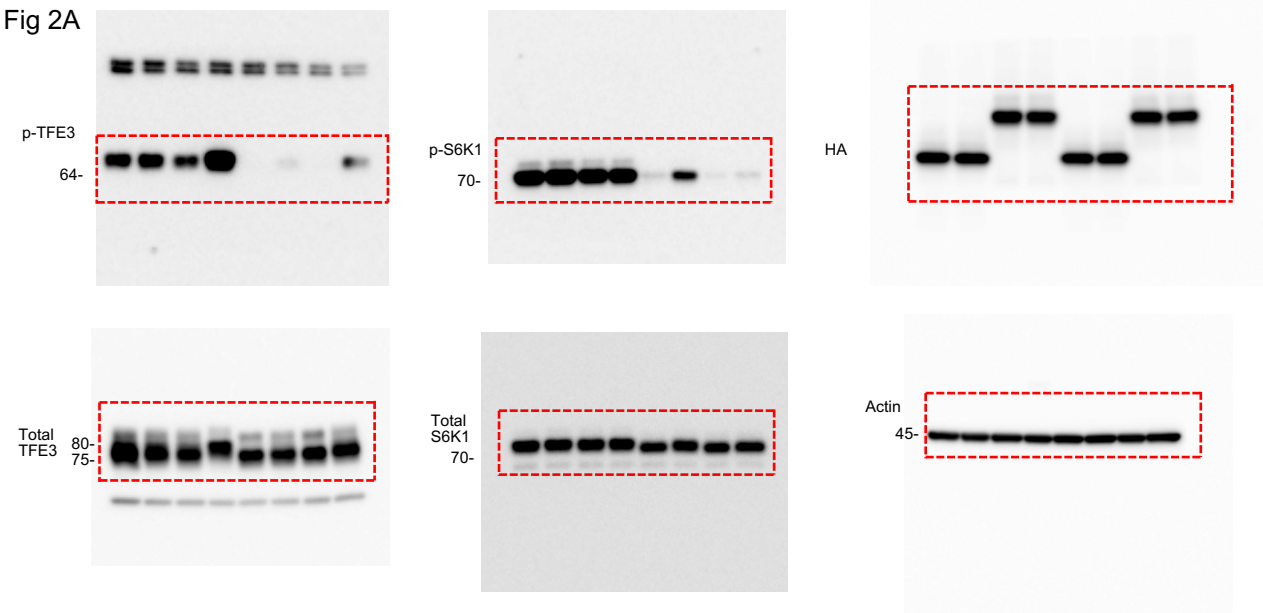

Fig 2C

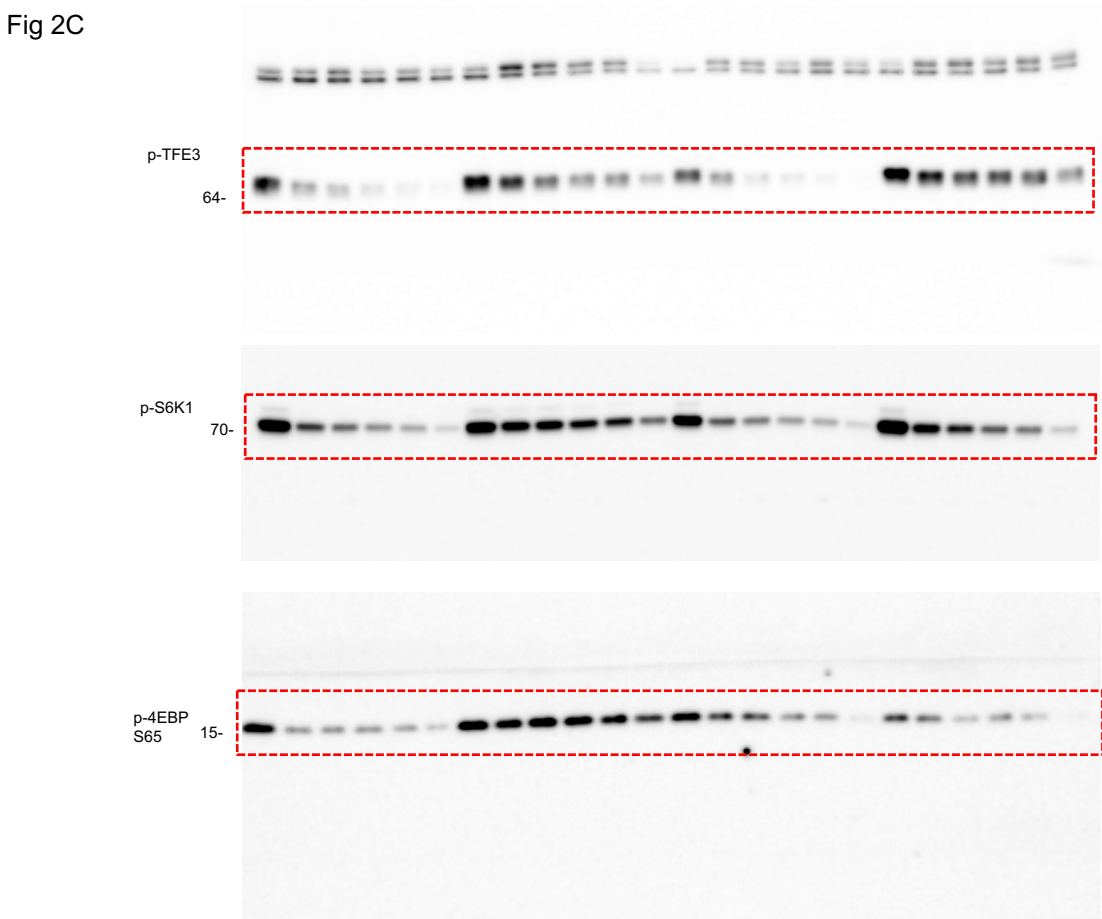

Fig 3A

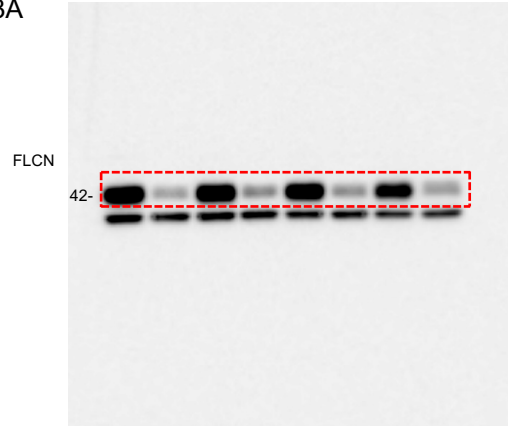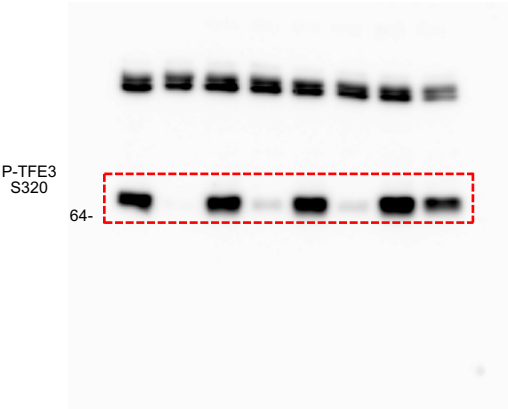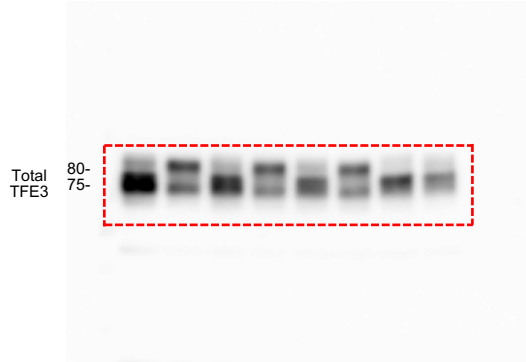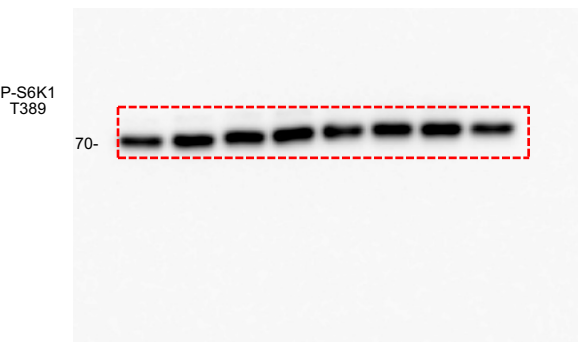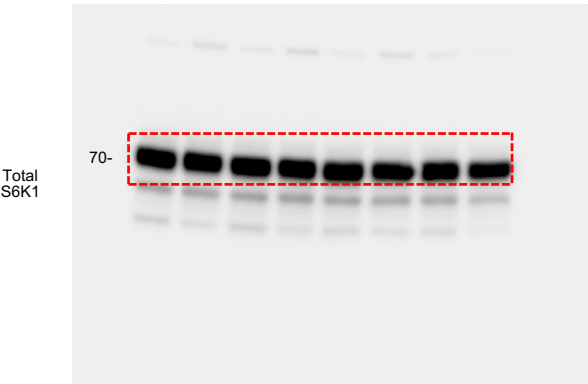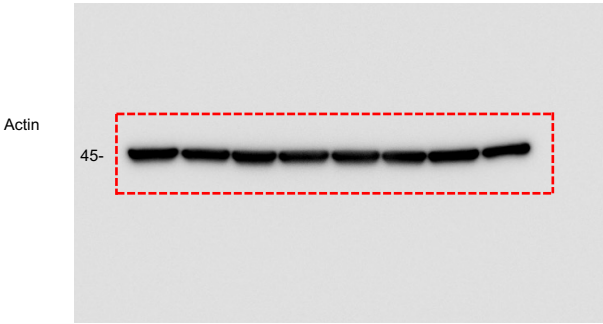

Fig 5A

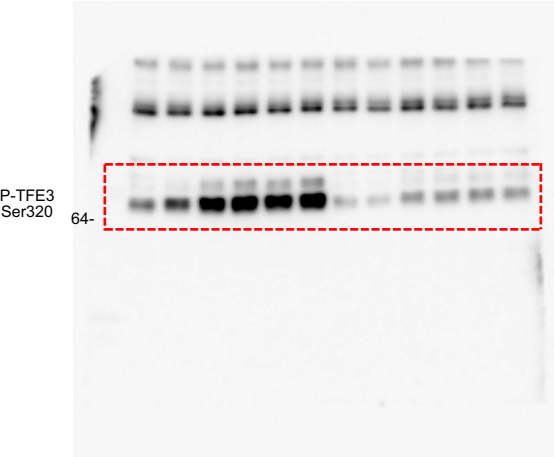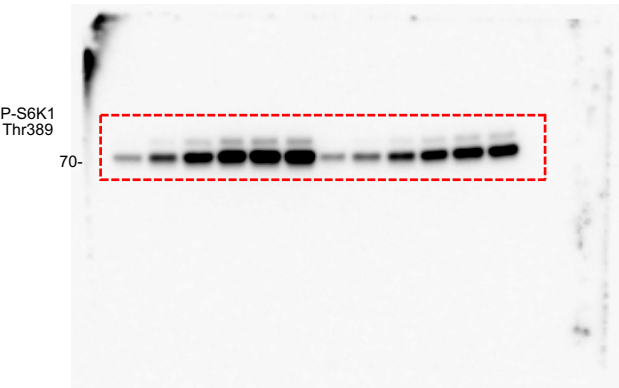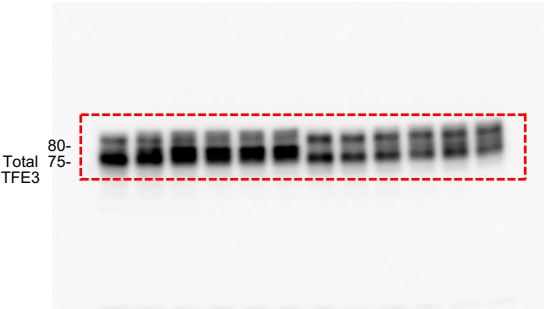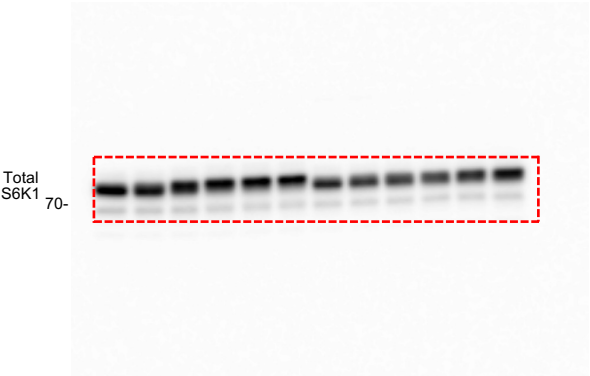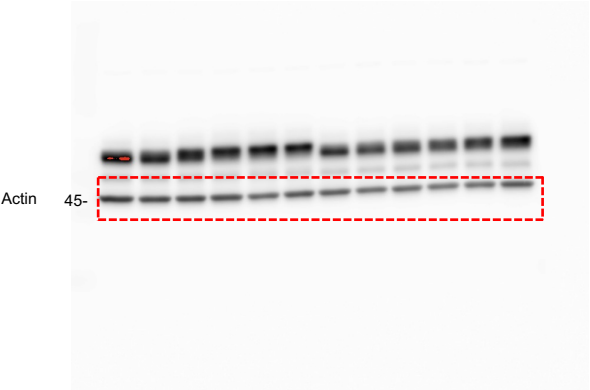

S2A

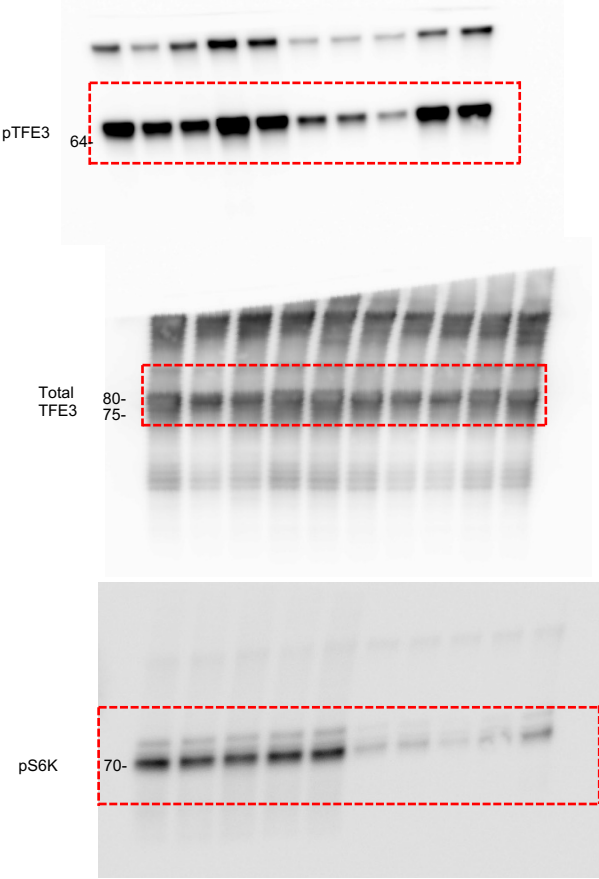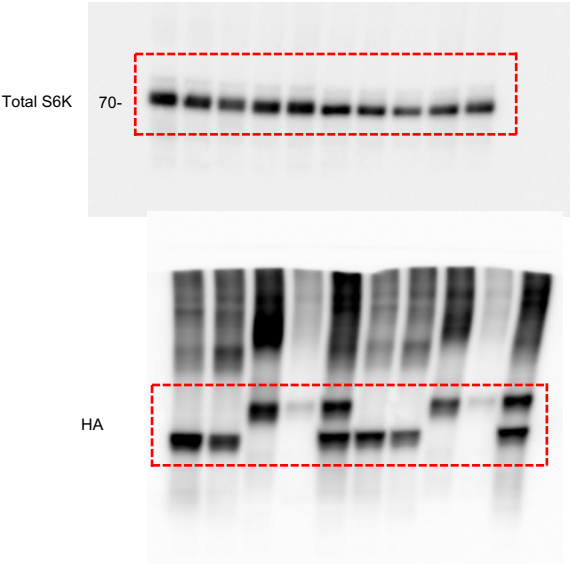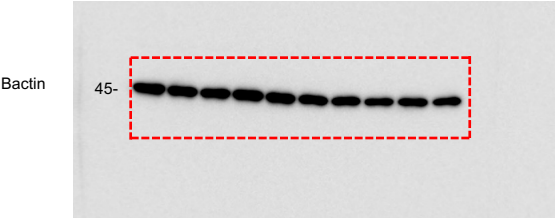

S2B

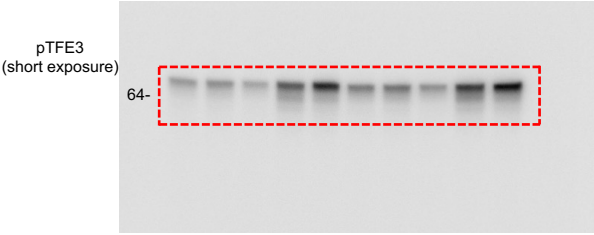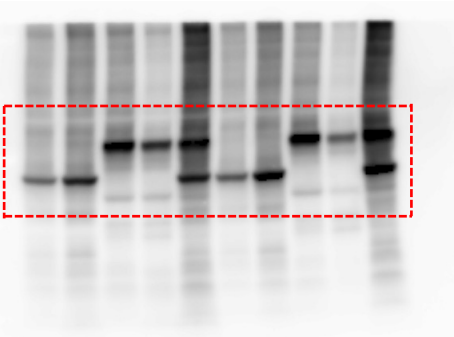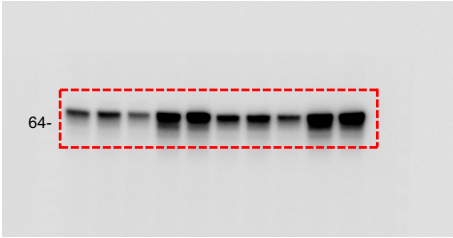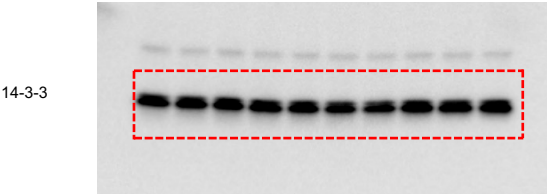

S2C

Total TFE3

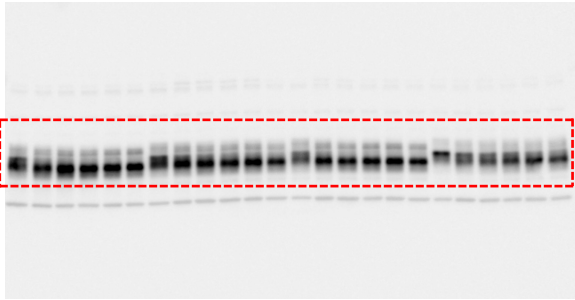

Total S6K1

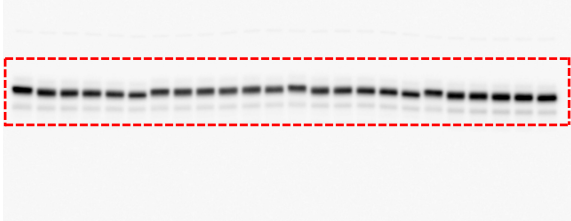

Total 4E-BP1

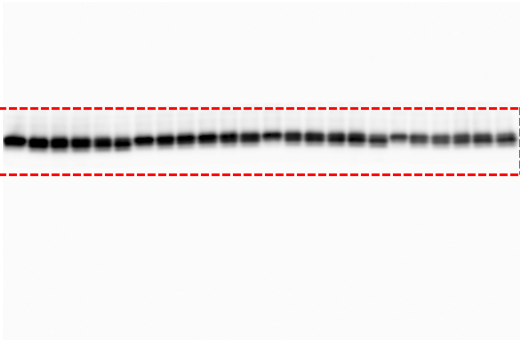

S3A

RagC

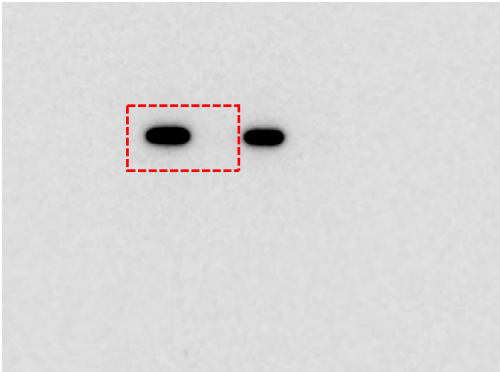

Actin

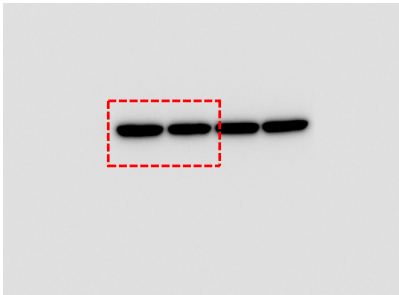

Supplement: S1 Raw Images — (PDF) [file pbio.3001594.s005.pdf]
