# Supplementary material for: Folliculin promotes substrate-selective mTORC1 activity by activating RagC to recruit TFE3
Source: PLoS Biol. 2022 Mar 31;20(3):e3001594. doi: 10.1371/journal.pbio.3001594 (PMC9004751; doi:10.1371/journal.pbio.3001594)
Supplement: Attachment — Submitted filename: response to reviewers.docx [file pbio.3001594.s006.docx]

We thank the reviewers for their interest in our work and their constructive input. We have addressed below in detail each point raised by the reviewers. We include numerous panels of new data, and we provide a tracked version of the manuscript to facilitate review.

**Reviewer 1**

This manuscript by Wada et al. describes how FLCN promotes TFE3 recruitment to the lysosome by activating RagC. TFE3 belongs to the MiT-TFE family of transcription factors, which also includes MITF, TFEB, and TFEC. Previous studies have shown that these transcription factors are regulated in a similar manner, through a mechanism that involves mTORC1-mediated control of nucleo-cytoplasmic shuttling. More recent studies by several groups, including the group of authors of the present manuscript, have shown that mTORC1-mediated regulation of TFEB and TFE3 occurs through a "non-canonical" pathway that requires the FLCN-RagC/D axis. In the present manuscript the authors show that an mTORC1 substrate-specific mechanism, which was previously shown for TFEB (PMID: 32612235), also operates for TFE3. This observation is interesting and potentially relevant, considering the evidence that these two transcription factors may have different roles and relevance in both physiological and
pathological settings. However, there are several issues, both in the text and figures, that need to be addressed by the authors.

CREDIT TO PREVIOUS STUDIES
The authors tend to ignore or minimize crucial contributions that other groups have previously made to the specific topic of the present manuscript. The following issues must be addressed by acknowledging previous discoveries and properly citing relevant previous papers.

1) A recent manuscript described a substrate-specific mechanism by which mTORC1 differentially phosphorylates its substrates and how this mechanism is relevant for Birt-Hogg-Dube' syndrome (PMID: 32612235). However, the results and conclusions of this previous manuscript have been largely ignored by the authors. Here are some examples: a) In the abstract (line 27) the authors stated "mTORC1 phosphorylates a myriad of substrates, but how different substrate specificity is conferred on mTORC1 by different conditions is largely unknown". This sentence does not take into account the mechanism recently described in ref. PMID: 32612235. b) Line 80. The hypothesis made by the authors at the end of the introduction is strikingly identical to the main conclusions of ref. PMID: 32612235. It looks like the authors ignored the important findings of this study by hypothesizing an identical mechanism… c) Line 291. The authors claim to have elucidated a new mechanism that enables
substrate-specific phosphorylation by mTORC1. However, once again, they seem to ignore that this mechanism was previously described for TFEB (PMID: 32612235). d) Lines 306-314. The "BHD mTORC1 hyperactivity paradox" was recently explained in reference PMID: 32612235 . e) Line 344. The sentence "The work mechanistically exposes the first example of parsing of mTORC1 signaling" is incorrect, as it does not account for the mTORC1 substrate-specific mechanism described in ref. PMID: 32612235.

We agree with the reviewer that our work in part reports similar findings to those of Ballabio et al. (PMID 32612235), as we had already noted in our discussion. We are well aware of the Ballabio study – in fact, we provided reagents for their study and helped guide them to their conclusions by communicating to them our results reported here. As suggested by the reviewer, we have improved our referencing of their work:

- We have changed the abstract line 27 to read “mTORC1 phosphorylates a myriad of substrates, but how different substrate specificity is conferred on mTORC1 by different conditions remains incompletely defined”
- We have added the following after line 80: “While the work that we report here was being finalized, the Ballabio group reported overlapping findings, with our input (ref: 32612235).”
- We have added the following after line 291: “similar findings were recently reported for the regulation of TFEB (ref: 32612235).”
- We have changed the last sentence of the discussion to read “Our work, combined with similar work with TFEB (ref: 32612235), mechanistically exposes the first clear example of parsing of mTORC1 signaling”.

2) Line 66. Although the authors were the first to report the evidence of a "non-canonical" mTORC1 pathway that mediates TFE3 phosphorylation in adipose and myeloid tissue, additional studies reported differential phosphorylation of TFE3 versus canonical mTORC1 substrates in FLCN deficient cells (PMID: 21209915, PMID: 31672913).

We have added to line 66 “…, subsequently also reported by the Zoncu group (ref: 31672913).

Ref 21209915 did not report data on canonical mTORC1 substrates, but we have added the reference in comment 3) below.

3) Line 110. The first evidence that FLCN regulates the phosphorylation of TFE3 was described in ref. PMID: 21209915. This reference should be added.

We have added to line 110 “…, as first described by the Linehan group (ref: 21209915).

4) Line 169. It has been well established by multiple studies that FLCN acts upstream of RagC (PMID: 24095279, PMID: 24081491, PMID: 31672913, PMID: 31704029). Such studies should be cited and the sentence "To test if RagC acts upstream or downstream of FLCN..." should be eliminated.

The phrase “acts upstream or downstream of FLCN” has been deleted, and the sentence shortened to its intended meaning: “To test if RagC confers specificity on noncanonical signaling to TFE3….”.

Refs 24095279, 31672913, 31704029 were referenced in the introduction (last paragraph)

Ref 24081491 has been added to the same paragraph: “… confirming earlier co-precipitation studies (ref: 24081491).

5) Line 177. The first evidence of an mTORC1-TFEB/TFE3-RagC/D feedback loop was reported in reference PMID: 28619945. Reference 7 only refers to the genes that are regulated by TFE3. Thus, reference PMID: 28619945 should be added when referring to the feedback loop (see also discussion Line 309).

The sentence has been modified to read: When activated and nuclear, TFE3 is known to drive a broad genetic program, including genes expressing lysosome proteins (*Mcoln1, Neu1, Hexa, Atp6v0e, Ctsa, Ctsb, Gpnmb*) (previous ref 7), regulators of mitochondrial biogenesis (*Ppargc1a*) (ref: 30843872), and a positive feedback loop to mTORC1 via *Ragd* (refs: 28619945, and ref3).

Ref 28619945 has also been added to discussion line 309.

TECHNICAL AND CONCEPTUAL ISSUES
6) Based on the data shown in Figure 2, the authors conclude that activation of RagA is required for the phosphorylation of S6K, but dispensable for the phosphorylation of TFE3. However, this conclusion cannot be drawn from the data shown in this manuscript for the following reasons: in Fig 2A the levels of TFE3 and S6K phosphorylation in starved cells expressing either active RagA or C are very weak. To claim that RagA activity is not relevant for TFE3 phosphorylation, the authors should compare the levels of TFE3 phosphorylation in cells expressing active RagC only, with those observed in cells expressing both active RagA and C. In Fig 2B a staining for RagA/C should be used to show which cells express active RagC/A. Furthermore, based on data in Fig 2B the authors claim that active RagC, but not active RagA, promotes cytosolic sequestration of TFE3 in the absence of AA. However, from these panels it appears to me that amino acid withdrawal induces TFE3 nuclear
translocation in both active RagA- and active RagC-expressing cells. The authors should also add quantifications for these IF data. In Figure 2C loading controls are missing, making quantifications unreliable. RagA/C immuno-blotting data are also missing. My overall impression from Fig 2C is that both active RagA and active RagC show a very similar effect on TFE3 phosphorylation in starved cells. In addition to the aforementioned technical issues, the statement that activation of RagA is dispensable for TFE3 phosphorylation conflicts with the data in Figure 1A showing constitutive phosphorylation of TFE3 in cells lacking DEPDC5, a specific inhibitor of RagA/B.

We apologize for the confusion – we do not conclude that activation of RagA is dispensable for the phosphorylation of TFE3. Our conclusion is that “RagA promotes canonical signaling to S6K and 4EBP in response to AA stimulation, while RagC promotes signaling to TFE3”, but this does not mean that RagA is dispensable for the latter. i.e., we find that RagA is not sufficient to confer activity on TFE3, but it may well be (and likely is) necessary. We have made this more clear in the text. Nevertheless, as suggested by the reviewer, we have also tested if the co-expression of active RagA and C confers further phosphorylation of TFE3 above RagC alone, and we find that it does not (new Supplemental Figure 2A), consistent with our conclusion that RagC, but not RagA, rescues TFE3 phosphorylation in the absence of FLCN. With respect to Figure 2B, the expression of active RagC/A was mediated by retroviral infection followed by selection for puromycin, which means that *all* cells express the indicated Rag. We have added quantifications for these IF data (new Figure 2B right panel), demonstrating the conclusion that active RagC, but not active RagA, promotes cytosolic sequestration of TFE3. With respect to Figure 2C, as requested by the reviewer, we have added RagA/C immunoblotting data (new Supplemental Figure 2B), demonstrating equivalent expression levels of these proteins at all time points. Finally, with respect to the DEPDC5 data in Figure 1, we again apologize for not making it clear that we are not concluding that activation of RagA is dispensable for TFE3 phosphorylation, but instead that it is not sufficient to do so. In fact, the DEPDC5 data support this conclusion, and we have added the following sentence to the text to make that point more clear: “The latter is consistent with the observation in Fig1D that activation of RagA, conferred by deletion of its inhibitor DEPDC5, is not sufficient to rescue TFE3 phosphorylation in the absence of FLCN.”.

New Supp Figure 2: RagC, but not RagA, promotes TFE3 phosphorylation in response to AAs. (A) Co-expression of active RagA and RagC does not confer further phosphorylation of TFE3 than active RagC alone. (B) To accompany main Figure 2C, immunoblotting for total levels of TFE3, S6K1, and 4E-BP in c2c12 cells expressing HA-tagged wildtype (WT), or constitutive active RagA (GTP) or RagC (GDP), demonstrate equivalent expression of these proteins at all time points after switching from complete medium to media lacking amino acids (AA).

New Fig 2B right panel: quantification

7) Line 221. The authors state that "RagC is dispensable for AA signaling to S6K". However, Rag GTPases are known to work as dimers of either RagA or B bound to RagC or D. No published evidence suggests that RagA/B could work as monomers. The authors' conclusion that RagC is dispensable for S6K phosphorylation is based on the data in Fig 5a showing that phosphorylation of S6K is only marginally affected in RagC-KO cells. However, RagC and RagD are well known to be able to compensate for each other. Therefore, in Fig 5A the authors should assess the levels of RagD (as well as those of RagC), to determine whether a possible RagD compensation mechanism exists in RagC-KO cells. In addition, to claim that RagC is dispensable for S6K phosphorylation, the authors should assess the phosphorylation of S6K in a RagC/D-double KO cell line or in RagC-KO cells silenced for RagD.

This is an interesting point, because indeed we have shown that loss of FLCN leads to dramatic induction of RagD (Li et al JCII 2019, and Figure 3C (consistent with the prior demonstration of TFE3/B-mediated induction of RagD, Di Malta et al 2017). We have therefore, as suggested by the reviewer, confirmed loss of RagC in the KO cells (new Supplemental Figure 3A) and checked to see if loss of RagC leads to compensatory induction of RagD, finding that it does so only very mildly in c2c12 cells and not at all in 293 cells (new Supplemental Figure 3B). Moreover, also as suggested by the reviewer, we show in new Supplemental Figure 3C that amino-acid induced phosphorylation of S6K can occur in cells in which both RagC and D are silenced by RNAi.

New Supp Figure 3: RagC AA-stimulated TFE3 phosphorylation is separable from canonical phosphorylation of S6K. (A) c2c12 cells with RagC CRISPR knockout were used for Figure5A. (B) Loss of RagC in c2c12 cells by CRISPR, or in 293Ts by siRNA, does not lead to significant compensatory induction of RagD. (C) 293T cells were silenced for RagC or RagC/D-double knockdown and maintained in media lacking amino acids for 60 minutes, and then returned to complete media for the indicated times.

8) Based on the data of Figure 6 the authors claim that anchoring TFE3 to different cytosolic compartments rescues its phosphorylation in FLCN-deficient cells. The mechanism by which such rescue occurs is unclear. In addition Figure 6 contains several important issues that need to be addressed (see below).Alternatively, the authors may consider eliminating this figure from the paper.

We believe that Figure 6 is important, especially in its surprising message that simply keeping TFE3 out of the nucleus is sufficient to promote its phosphorylation. We have addressed point by point the comments raised by the reviewer. However, if the reviewer and editor feel that it would be best to eliminate this figure, we would defer to their judgement.

Points to be addressed in Figure 6:
Fig6A: the levels of TFE3-Rheb15 in FLCN-KO cells are huge compared to the levels of either WT-TFE3 or TFE3-CAAX observed in the same cells (compare lane 4 with lanes 2 and 6). With such a huge difference in expression levels, it is impossible to come to any conclusion on the phosphorylation status of the different constructs in FLCN-KO cells.

Our western blots are developed using luminescence and thus in the linear range well beyond that appreciated by the naked eye. To quantify these results, we have repeated this experiment three times, with exactly the same results (new images and quantification in Fig6A, and images in new Supplemental Figure 4A), clearly demonstrating that fusing Rheb15 to TFE3 partially prevents the loss of its phosphorylation after FLCN deletion, in a manner dependent on its CAAX domain.

Figure 6A

New Supplemental Figure S4A: Repeat experiments of Fig 6A, where control c2c12 cells and cells lacking *Flcn* (KO) were transduced with HA-tagged wildtype TFE3, or TFE3 fused to Rheb15 lysosome-targeting sequence, or the same sequence in which the CAAX domain was deleted, followed by HA immunoprecipitations and western blotting as indicated.

Fig6E: Similarly, the levels of deltaNLS-TFE3 in FLCN-KO cells are massively higher than the levels of WT-TFE3 in the same cells (compare lane 6 with lane 2). Once again, it is impossible to make any comparison between these two samples.

Thank you for pointing this out. Suspecting an artifact of transfection, we repeated the experiment in triplicate and find in the new experiments that the levels of WT-TFE3 are no longer reduced in FLCN-KO cells, allowing for better comparison between WT and delta-NLS-TFE3 (new Supplemental Figure 4B). We also provide quantification of the results. Our conclusion from this experiment remains unchanged, i.e. that preventing TFE3 access to the nucleus prevents the loss of FLCN to promote TFE3 dephosphorylation.

New Supplemental Figure S4B: Repeat experiments of 6D, in which control c2c12 cells and cells lacking *Flcn* (KO) were transduced with HA-tagged TFE3 WT or ∆NLS, followed by immunoblotting for FLCN, TFE3 (HA), and phospho-TFE3. Ratio of pTFE3:HA signal intensity is shown on the right.

Fig 6D: The authors need to compare on the same blot the phosphorylation of TFE3 fusion proteins in WT and FLCN-KO cells with the phosphorylation levels of WT TFE3 in the same cells. Are these fusion proteins efficiently phosphorylated compared to WT TFE3 in control cells? Is there any difference in the phosphorylation of WT vs chimeric TFE3 (expressed at similar levels) in FLCN-KO cells? Without this comparison it is impossible to assess if there is any "rescue" of TFE3 phosphorylation by linking it to specific cellular compartments in FLCN-KO cells.

Our focus here was to test the response of phosphorylation of each construct in response to FLCN deletion, not to compare the phosphorylation level of the constructs to each other. We have made this more clear in the text, and removed the word “rescue” as we agree with the reviewer it was not our intent to make claims about rescue, but rather about the effects of FLCN deletion on each construct. We have also repeated the experiments in triplicate (new Supplemental Figure 5A,B), and quantified the blots (New Figure 6C right panel).

New Figure 6C quantification

New Supplemental Figure S5A-B: c2c12 cells were transduced with HA-tagged TFE3 fused to Tmem192 lysosome-targeting, Rab1a golgi-targeting, or Sec61b ER-targeting sequences, followed by immunoblotting and quantification as indicated.

Fig6B-C: the localization of TFE3-Rheb15 appears diffuse with no evident colocalization of TFE3 with either lysosomes, Golgi or ER. Similarly, I cannot appreciate any co-localization between TFE3-Tmem192 with LAMP2 (lysosomes), or between TFE3-Sec61b and PDI (ER). I suggest to perform higher quality IF analysis for co-localization of the chimeric protein with the specific cellular compartments and to perform, in addition, biochemical fractionation of the different organelles to confirm these data.

With respect to the localizations of TFE3-Rheb15, TFE3-Tmem192, and TFE3-Sec61b, we agree with the reviewers that the co-localization is not perfect. We have tempered these claims from the text (and moved the images to supplemental information), and focused on the main point of this series of experiments, i.e. that exclusion from the nucleus suffices to keep TFE3 phosphorylated, even in the absence of FLCN.

9) Related to point 7), the title of the paragraph "RagC, but not RagA, rescues TFE3 phosphorylation in the absence of FLCN" (lane 168) is misleading. The authors may want to change it with "Active RagC rescues TFE3 phosphorylation in the absence of FLCN, while active RagA does not".

This has been done.

10) The levels of total TFE3 in Fig 1A seem to vary considerably in the different treatments/cell lines. The authors should quantify their data and the levels of pTFE3 should be normalized with total TFE3.

This has been done (new Supplemental Figure 1)

New Supplemental Figure S1: quantification of Figure 1A. TFE3 phosphorylation is responsive to amino acids, via the GATOR complex. Control c2c12 cells (NTC) or cells lacking Flcn, Tsc2, or Depdc5, were switched from complete medium to media lacking serum and/or amino acids for 60 minutes followed by immunoblotting. Images were uploaded into ImageJ and signal intensity was quantified. Graphed is the ratio of p-TFE3 to total TFE3 signal.

11) Figure 3: In panel A the authors should clearly label WT and FLCN-KO cells; in panel B a staining for RagA/C should be used to show which cells express active RagC/A. Panel C: Due to the huge difference in the expression levels of TFE3 targets in WT and FLCN-KO cells, the graph in Fig 3C is hard to visualize. The transcript levels of TFE3 targets should be represented as fold change of mRNA levels in FLCN-KO cells VS NTC or the authors should separate the data of the two cell lines to allow a better comparison.

Panel A: this has been done. Panel B: all cells express active RagC/A because the Rags were delivered by retrovirus followed by selection. Panel C: we have regraphed the data for easier visibility. All data are now represented as fold change of mRNA in FLCN-KO vs NTC cells.

12) Figure legends lack the information regarding the statistics for each experiment and the number of times (n) experiments were performed. In addition, scale bars are missing in Fig 1, Fig 2, Fig 3, Fig 5 and Fig 6.

This has been done.

**Reviewer 2**

It was previously shown that mTORC1-dependent phosphorylation of the transcription factor TFE3 is lost in FLCN deficient cells. Here, the authors study the mechanism of TFE3 phosphorylation by mTORC1. In response to amino acids, FLCN activates RagC, which in turn recruits TFE3 to the lysosome. The authors show that in FLCN or RagC deficient cells, TFE3 is active in the nucleus. This phenotype is suppressed by overexpression of constitutively active RagC. Phosphorylation of the mTORC1 substrate S6K is unperturbed in FLCN or RagC deficient cells. Finally, the authors claim that by expelling TFE3 from the nucleus, by forcing localization to the lysosome, ER, Golgi or cytoplasm, TFE3 phosphorylation is restored even in the absence of FLCN. Specific comments are as follows.

1) The authors study TFE3 phosphorylation in cells cultured in different media: full medium, amino acid deficient, serum deficient and lacking both amino acids and serum. Based on this, the authors claim that growth factor signaling is dispensable for TFE3 phosphorylation (lines 105 -106 and 134), despite the fact that mTORC1 is normally growth factor dependent. Nevertheless, in Fig.1A TFE3 phosphorylation is reduced in serum starved cells compared to full medium. Also, surprisingly, in TSC2 deficient cells, TFE3 phosphorylation is reduced in all conditions. How do the authors explain these phenotypes? The authors should provide an explanation for the loss of TFE3 phosphorylation upon serum starvation (if mTORC1 is not the TFE3 kinase). They should also provide a more compelling explanation for why TFE3 phosphorylation is reduced in TSC2 deficient cells. In general, there are contradictory and confusing claims on the role of mTORC1 in TFE3 phosphorylation.

We did not mean to claim that “growth factor signaling is dispensable for TFE3 phosphorylation”, but rather that growth factor signaling *via TSC inhibition* cannot promote phosphorylation of TFE3. There may well be other pathways for growth factor signaling to TFE3, although we do not see much evidence of that in our studies. With respect to Figure 1A, please note that the total amount of TFE3 is reduced in the absence of growth factors (as is the total amount of S6K), so that the ratio of phosphorylated TFE3 is only partially reduced compared to the presence of growth factors, and much less so than in the absence of amino acids (see quantification in new Supplemental Figure 1A). The astute question raised by the reviewer of why phosphorylation of TFE3 declines in TSC2KO cells is fascinating, and suggests the existence of a negative feedback loop, whereby activation of canonical mTORC1 signaling feeds back to suppress non-canonical signaling, perhaps via suppression of FLCN itself. We are actively investigating this possibility. Finally we do want to make clear that we do think that mTORC1 is the kinase for this phosphorylation site of TFE3, as we have demonstrate previously with purified components (Wada et al. Genes & Development 2016).

New Supplemental Figure S1: quantification of Figure 1A. TFE3 phosphorylation is responsive to amino acids, via the GATOR complex. Control c2c12 cells (NTC) or cells lacking Flcn, Tsc2, or Depdc5, were switched from complete medium to media lacking serum and/or amino acids for 60 minutes followed by immunoblotting. Images were uploaded into ImageJ and signal intensity was quantified. Graphed is the ratio of p-TFE3 to total TFE3 signal.

2) The experiments were performed exclusively in C2C12 cells. Do their findings apply to other cell lines?

Yes. We provide new data (Supplemental Figures 2A and 3C) showing similar data in 293 cells, and also refer to the recently published work by the Ballabio group using HeLa FLCN KO cells which we provided to them (Napolitano et al. Science 2019).

New Supplemental Figure S2A: RagC, but not RagA, promotes TFE3 phosphorylation in response to AAs. (A) Co-expression of active RagA and RagC in 293T cells does not confer further phosphorylation of TFE3 than does active RagC alone.

New Supplemental Figure S3C: 293T cells were silenced for RagC or RagC/D-double knockdown and maintained in media lacking amino acids for 60 minutes, and then returned to complete media for the indicated times.

3) Fig. 6. The authors force TFE3 localization to the cytoplasm, ER, Golgi or lysosome. Under these conditions, the authors claim that TFE3 is phosphorylated by mTORC1 even in the absence of the mTORC1 activator FLCN. How do the authors explain this observation?

Our data suggest that increasing resident time of TFE3 outside the nucleus likely gives it access to mTORC1 in any number of different locales, which is sufficient to promote TFE3 phosphorylation.

4) Regarding the concluding statement in lines 341-342, the author claim to "elucidate the mechanistic basis by which FLCN confers substrate specificity upon the mTORC1." This statement is too strong. There are still open questions on the exact regulation (see point 1). Moreover, the authors fail to mention a publication from the Ballabio group (G. Napolitano et al, Nature, 2020) claiming a substrate-specific mechanism of TFEB phosphorylation by mTORC1. The authors should rephrase their concluding statement.

We have added reference to the work by the Ballabio group to the concluding sentence.

5) The authors claim that S6K phosphorylation is unperturbed by deletion of the FLCN gene. However, it has been shown that in FLCN deleted cells S6K phosphorylation is either lost or increased compared to control (e.g. Z. Tsun et al, Molecular Cell, 2013 and Y. Hasumi et al, Human Molecular Genetics, 2014). How can these different phenotypes be explained by the authors' findings? On a similar note, the authors claim that RagC deletion has no effect on S6K phosphorylation. However, the James group (e.g. G. Yang et al, EMBO, 2018) showed a reduction in S6K phosphorylation in RagC shRNA treated cells. The manuscript would benefit from inclusion of these two points the Discussion.

We also have noted the difference between our findings and those of Tsun et al. However, we note that we have made the same observation in numerous other cell types including adipocytes (Wada et al. G&D 2016) and monocyte/macrophages (Li et al. JCII 2019), and other groups have also reported that S6K phosphorylation occurs in the absence of FLCN, including the Ballabio group (see point 2) above). One difference may lie in our use of complete KO via CRISPR, in contrast to siRNA approach taken by Tsun et al., which may have differences in, for example, chronic vs subacute knockdown. The observation that in some contexts S6K phosphorylation is in fact elevated is quite interesting, and we have reported on it in recent work (Li et al. JCII 2019): the explanation likely lies in the fact that in some cells, such as monocytes and likely the cardiomyocytes in Hasumi et al., a feedback loop is activated in the absence of FLCN whereby TFE3 promotes the strong transcriptional induction of RagD, which then promotes mTORC1 activity on S6K. This feedback loop is discussed in the second paragraph of the discussion. Finally, with respect to the excellent work by Yang et al., the majority of data investigating effects on S6K in their work uses overexpression of RagC mutants, likely working in a dominant-negative fashion. A single figure in supplemental data (EV2) provides data to suggest that RagC shRNA partially suppresses S6K phosphorylation in HEK-293E cells. However, in the absence of replicated experiments, and of quantification of the pS6K/S6K ratio (it can be seen in the figure that total S6K is less abundant), it is difficult make firm conclusions.

6) Related to statement of lines 196-197, the authors use Torin to increase intracellular amino acids level while blocking mTORC1 activity. The authors should explain how Torin, an mTORC1 and mTORC2 inhibitor, increases nutrients in cells.

We apologize for the confusion, we did not use Torin1 to increase intracellular amino acids, but rather to “capture” the transient translocation to the lysosome of mTORC1, because inactive mTORC1 appears unable to escape the lysosome once activated.

7) Fig. 3C. Graphs in this image lack error bars.

This has been done.

8) Introduction. The Introduction would benefit from more citations. Lines 48-52 and 56-64 completely lack references where some should be provided.

Additional citations to excellent reviews on mTOR biology have been provided.

9) Figures. To improve clarity, the authors should always add treatment information (e.g., drug concentration, time and media condition) in figure legends. Figures should be self-explanatory. For example, in Fig. 6 the authors should indicate which gene is KO'ed (FLCN). Moreover, it is difficult to distinguish colors in dot bar graphs. The authors should make graphs clearer. Finally, the authors should add axis labels to Fig. 1C and Fig. 2C.

This has been done.

10) The blots are generally over-exposed which might mask important differences. The total TFE3 antibody gives a different pattern in Fig 2A.

Our western blots are developed using luminescence and thus in the linear range well beyond that appreciated by the naked eye. We have provided new quantifications throughout the manuscript to support the visual data.
