# Supplementary material for: Folliculin promotes substrate-selective mTORC1 activity by activating RagC to recruit TFE3
Source: PLoS Biol. 2022 Mar 31;20(3):e3001594. doi: 10.1371/journal.pbio.3001594 (PMC9004751; doi:10.1371/journal.pbio.3001594)
Supplement: Attachment — Submitted filename: response to reviewers.docx [file pbio.3001594.s007.docx]

REVIEWER 1:

1. INDEPENDENCE OF S6K PHOSPHORYLATION FROM THE PRESENCE OF RAGC/D
Based on the data shown in new Supplementary Figure 3, the authors claim that S6K can be phopshorylated by mTORC1 in the absence of RagC/D. However, as I had already pointed out in the first revision of this manuscript (point 7), a clear distinction should be made between the presence and the activity of Rag C/D GTPases. While the authors convincingly show that the activity of RagC/D is dispensable for the phosphorylation of S6K, the data shown in this manuscript are too weak to claim that S6K can be phosphorylated in the absence of RagC/D. Furthermore, there is a vast and robust literature showing that Rag GTPases can only work as heterodimers of RagA/B bound to RagC/D. No evidence so far has ever shown or suggested that RagA/B can work as monomers. The possible discovery that RagA/B can activate mTORC1 in the absence of RagC/D would be a revolutionary finding in the field and, therefore, would need to be unequivocally demonstrated with biochemical, structural, and
functional analyses. If true, this would be the focus of an entirely different manuscript in which the authors would need to show how RagA/B monomers are able to activate mTORC1. In any case, I remain very skeptical about this possibility as I find the data shown in this manuscript too weak to make such a claim. Therefore, I suggest removing the data on RagC depletion and to keep the data obtained using active RagC, which explore the activity, rather than the presence, of RagC.

We agree with the reviewer and have made edits in the text to make clear that the message is concordant with the reviewer’s points. Specifically: we show unequivocally, using CRISPR KO cells, that S6K can be phosphorylated in the absence of RagC (Fig 5A), but we agree with the reviewer that this does not demonstrate that RagD is dispensable. We do show that there is no compensatory increase in RagD expression in the cells lacking RagC (Fig S3C, now S3B), demonstrating that the preexisting amount of RagD present is sufficient to allow efficient phosphorylation of S6K in the absence of RagC. At the suggestion of the reviewer, we have removed the RagD siRNA experiments (old Fig S3B) – we can reintroduce these data if the editors or reviewer would find it more appropriate.

2. INDEPENDENCE OF TFE3 PHOSPHORYLATION FROM THE ACTIVITY OF RAGA/B
In response to my concerns in point 6 of the rebuttal, the authors state that they "do not conclude that activation of RagA is dispensable for the phosphorylation of TFE3" and that "RagA is not sufficient to confer activity on TFE3, but it may well be (and likely is) necessary". While I agree with these statements, the authors also state several times in the manuscript that the activity of RagC is sufficient to promote TFE3 phosphorylation. These two concepts contradict each other. The concept that RagC activation is sufficient to induce TFE3 phosphorylation of course implies that the activity of RagA is dispensable, which is in contrast with the authors' own data and statements. Therefore, the authors should fix these issues and re-phrase their claims about the role of the activity Rag GTPases, specifying that while the activity of RagA is essential for the phosphorylation of both TFE3 and S6K, the activity of RagC is important only for the phosphorylation of TFE3.

We have edited the text to make these distinctions more clear: what we mean to claim is that activation of RagC is sufficient to promote TFE3 phosphorylation *without simultaneous additional activation of RagA/B (*Fig 2). We agree with the reviewer that RagA is unlikely to be dispensable, and do not make that claim.

3. CYTOPLASMIC LOCALIZATION OF TFE3 IS SUFFICIENT TO RESCUE ITS PHOSPHORYLATION IN THE ABSENCE OF FLCN
The authors failed to address my previous concerns on Figure 6. The data contained in this Figure are too weak to support the authors' claim. Figure 6 contains several issues (e.g. a huge difference in the expression of the different proteins analysed, lack of proper localization of the chimeric proteins, inconsistency of data among different panels) and no conclusions can be drawn from these data. Importantly, no mechanism is provided to explain why TFE3 phosphorylation would be rescued in FLCN-KO by simply forcing its localization to different cytoplasmic compartments. This message is in sharp contrast with the overall message of the manuscript (i.e. inactivation of RagC drives TFE3 de-phosphorylation in FLCN-KO cells). Finally, forcing the localization of TFE3 in several cytoplasmic structures (such as Golgi, ER etc…) does not address the "recruitment of TFE3 to mTORC1", as stated in the title of this section. Thus, in my opinion this section, including Figure 6, should
be removed from the manuscript.

We agree with the reviewer that the data shown in Figure 6 do not fit easily in current dogma on how the mTORC1 complex functions. We also agree with the reviewer the data in this figure are not necessary to make the overall message of the manuscript, and thus we are willing to remove the figure entirely. However, we do think that these data are interesting and provocative and thus worthy of being shared with the scientific community, and we thus propose the attached edits, in which we substantially temper conclusions drawn from the data. If, after reviewing these edits, the reviewer and editor still feel it to be more appropriate to remove the figure altogether, we can do so.


REVIEWER 2:

The authors addressed all concerns raised previously. Specific comments are as follows:

1) Related to previous comment 1. Lines 136-139 (before 132-135) state "Together, these data demonstrate that the presence of AAs is necessary and sufficient to promote phosphorylation of TFE3 by mTORC1, and does so via GATOR1 and FLCN, while growth factors are largely dispensable, in sharp contrast to canonical phosphorylation of S6K." The authors should rephrase as this sentence is misleading. The authors should instead include part of the explanation provided to the reviewer, i.e., that the absence of TSC2 is not sufficient to promote TFE3 phosphorylation.

This has been done

According to the Supplementary Figure 1A, in untreated cells TFE3 phosphorylation is reduced by over 60% in serum-starved conditions compared to full media conditions, indicating at least a partial regulation of TFE3 phosphorylation by growth factors. If the authors want to conclude that S6K phosphorylation dependents more on growth factors than TFE3 phosphorylation, a quantification of S6K phosphorylation of Figure 1A (similarly to Supplementary Figure 1A) should be provided.

This has been done.

2) Regarding comment 2. The authors should explain in the text why in amino acids-starved 293T cells the expression of RagA-66L is not sufficient to promote S6K phosphorylation as shown in Figure 2A for c2c12 cells. Moreover, the author should repeat the experiment in Supplementary figure 2A with equal expression of RagC-WT and RagC-75L. Finally, Figure 2A and Supplementary Figure 2A have different annotations (GDP/GTP vs. 66L/75L), the authors should correct it and keep the same nomenclature in all figures.

We have repeated the experiment in Supplementary figure 2A and again find that expression of RagC-75L is much lower than that of WT. Because the amounts of plasmid used for transfection are identical, we suspected that RagC-75L protein may be less stable than WT. We therefore repeated the experiment in the presence of proteasome inhibition by MG 132 (new Supplementary figure 2B), and indeed, more RagC-75L protein was now visible. Importantly, both in the absence and presence of MG132, phosphor-TFE3 is abundant, indicating that even reduced amounts of RagC-75L are sufficient to promote phosphorylation of TFE3, underscoring the main message of the manuscript. With respect to RagA-66L effect on S6K phosphorylation, we think the difference is likely ascribed to the different cells used in the two experiments. Finally, we have corrected the annotations and nomenclature for GDP/GTP (instead of 66L/75L) throughout the manuscript, as suggested.

3) Supplementary Figure 3C and lines 232-234. The authors' conclusion is not supported by the data. The authors should provide quantification as it appears that phosphorylated S6K is increased upon RagC and RagC/D RNAi treatment. Also, the author should blot phospho and total TFE3 for the same condition (and quantify) as well as RagC and RagD to confirm proper knock down.

At the request of Reviewer 1, Supplementary Figure 3C has been removed.

4) Regarding comment 5. It would be helpful if the authors included in the Discussion at least part of the explanation provided to the reviewer.

This has been done

5) Regarding comment 8. The author should add references for lines 48-52 and 56-63.

This has been done

6) Regarding comment 9. It would facilitate the readers' understanding if the authors indicate which gene is KO'ed (FLCN) in the following figures: 3A, S3A, S4A (both blots), S4B, S5A, S5B (all three blots), 6A, 6C and 6D

This has been done
