# Supplementary material for: Folliculin promotes substrate-selective mTORC1 activity by activating RagC to recruit TFE3
Source: PLoS Biol. 2022 Mar 31;20(3):e3001594. doi: 10.1371/journal.pbio.3001594 (PMC9004751; doi:10.1371/journal.pbio.3001594)
Supplement: Attachment — Submitted filename: Response to reviewers.docx [file pbio.3001594.s008.docx]

Editors’ comments

Reference 13 should be added in the context of line 66 - “We have recently identified a substrate-specific branch of mTORC1 signaling, providing the first example of specific regulation of different branches of mTORC1 signaling [6, 7], subsequently also reported by the Zoncu group [8]” - as the title of that reference states "A substrate-specific mTORC1 pathway". Please also rephrase the last sentence of the Introduction by deleting 'with our input' to avoid misunderstandings: "While the work that we report here was being finalized, the
Ballabio group reported overlapping findings with TFEB, with our input [13]".

This has been done

We would like you to consider the following suggestion [for title]: “Folliculin promotes substrate-selective mTORC1 activity by activating RagC to recruit TFE3”

This has been done

Reviewers' comments

Rev. 1:
The authors have satisfactorily addressed some of my criticisms. However, the remaining points are still problematic and need to be properly addressed:

1) As previously stated by this reviewer, data in Figure 6 are very weak and not suitable for publication. The main problems of this figure are: huge difference in the expression of the different proteins analysed, lack of proper localization of the chimeric proteins and inconsistency of data among the different figure panels. Thus, in my opinion Figure 6 needs to be removed from the manuscript.

figure 6 has been removed, as well as related supplemental figures S4-6.

2) The authors did not properly addressed point 2 of my report: INDEPENDENCE OF TFE3 PHOSPHORYLATION FROM THE ACTIVITY OF RAGA/B. While the authors state that they agree with this reviewer and that RagC is NOT sufficient for TFE3 phosphorylation, as RagA activity is in fact necessary for this process, they now state that "RagC is sufficient to promote TFE3 phosphorylation without simultaneous additional activation of RagA/B". This would imply that Rag GTPases can recruit and phosphorylate TFE3 even when RagA/B are inactive. This is wrong, as it is well established that activation of RagA/B is required for mTORC1 lysosomal recruitment and activation. The text should be changed accordingly.

The sentence has been amended to now read: “These data thus demonstrate clearly separable Rag-mediated pathways, whereby RagA is sufficient to promote canonical signaling to S6K and 4EBP in response to AA stimulation, while RagC is sufficient to promote signaling to TFE3 *without simultaneous additional activation of RagA beyond its preexisting baseline activity*.” (italics added only here).
